# Clinician Diagnostic Ratings and Countertransference Reactions Towards Grandiose and Vulnerable Narcissism

**DOI:** 10.1002/cpp.70070

**Published:** 2025-03-27

**Authors:** Nicholas J. S. Day, Marko Biberdzic, Ava Green, Georgia Denmeade, Bo Bach, Brin F. S. Grenyer

**Affiliations:** ^1^ School of Psychology University of Wollongong Wollongong Australia; ^2^ Department of Psychology Bishop's University Sherbrooke Canada; ^3^ Department of Psychology City St George's, University of London London UK; ^4^ Center for Personality Disorder Research Psychiatric Research Unit, Mental Health Services Region Zealand Denmark; ^5^ Department of Psychology University of Copenhagen Copenhagen Denmark

**Keywords:** countertransference, diagnosis, grandiosity, narcissistic personality disorder, vulnerability

## Abstract

**Background:**

Narcissistic personality disorder (NPD) is known to comprise two distinct but connected phenotypes related to ‘grandiosity’ and ‘vulnerability’, respectively. While evidence suggests differing countertransference responses to narcissism subtype expression, no study has examined this using a qualitative methodology and explored associations with ratings of personality disorder severity.

**Method:**

Mental health clinicians (*N* = 180, 67% female, age = 38.9), completed qualitative clinical reflections and ratings of overall personality disorder severity towards two hypothetical vignettes displaying pathological narcissism (‘grandiose’ and ‘vulnerable’ narcissism respectively), as well as a rating of attitudes towards patients in their routine practice who resemble these vignettes.

**Results:**

Distinct qualitative themes were identified between narcissistic subtype, with grandiose narcissism evoking anger, lack of empathy and hopelessness, compared to sympathy, sadness and discomfort in vulnerable narcissism. In terms of diagnostic category, the grandiose vignette was predominately identified as ‘narcissistic personality disorder’ (97%), whereas the vulnerable vignette was a mixture of ‘depressive disorder’ (29%), ‘narcissistic personality disorder’ (24%), ‘trauma and stressor related disorders’ (21%) and ‘borderline personality disorder’ (21%). Attitude scores differed significantly between subtypes, with more negative attitudes towards narcissistic grandiosity than narcissistic vulnerability. The grandiose vignette was also rated as displaying more overall personality impairment, with an association observed between negative clinician attitude scores and increased ratings of personality disorder severity.

**Discussion:**

Two potential pathways are outlined to interpret these findings. The first is that inordinate stigma towards narcissistic grandiosity negatively biases clinicians when working with these patients due to feelings of anger and frustration. The second is that clinicians are drawn to minimise pathology of vulnerable patients due to their feelings of sadness and empathy. We propose that effective diagnosis and psychotherapy for pathological narcissism rely on clinicians' ability to balance these two dilemmas and resist either extreme.

## Summary

- Narcissistic pathology can stir up intense and contradictory emotions in treating clinicians, including frustration and feelings of incompetence, as well as sadness and empathy.
- Such strong countertransference may impair clinician diagnostic efforts, risking negative reactions and inordinate stigma towards such patients, or alternatively offering an ineffective superficially supportive treatment.
- Effective treatment of pathological narcissism involves resisting the extremes of particular interpersonal ‘pulls’ (e.g., to defensively criticise, blame or dismiss; or to excessively empathise, encourage and deny their contribution to their difficulty).
- Clinician countertransference may also serve as a useful cue as to the primary unconscious emotional states and preoccupations (e.g., grandiosity, self‐loathing) that exist within the patient.

## 1 Introduction

Narcissistic personality disorder (NPD) is a severe and complex mental health condition involving an unrealistically inflated sense of self and challenging interpersonal patterns that put one at odds with others (American Psychiatric Association 2013). While diagnostic criteria for NPD contain many important features relevant to the disorder, the criticisms of the narrow focus of grandiose features to the exclusion of more vulnerable ones is well documented (Cain et al. 2008). As such, the term ‘pathological narcissism’ is frequently used as a way to describe narcissistic phenomena that encompasses both grandiose and vulnerable states (Pincus et al. 2009; Pincus and Lukowitsky 2010). Grandiose elements include instances of fantasies of omnipotence, self‐importance and admiration‐seeking, whereas vulnerable elements include instances of interpersonal hypersensitivity, feelings of shame, victimhood and affective instability (Day et al. 2020). While clinical patients typically present in predominately one of these subtypes, grandiose and vulnerable elements are interrelated (Krizan and Herlache 2017) and often oscillate over time (Gore and Widiger 2016; Oltmanns and Widiger 2018). However, while the expression of narcissism may differ, both grandiose and vulnerable elements are marked by interpersonal difficulties (Dashineau et al. 2019; Dickinson and Pincus 2003; Edershile and Wright 2019), which includes the interpersonal situation of the therapeutic relationship (Kealy and Ogrodniczuk 2011; Kernberg 2014; Weinberg and Ronningstam 2022).

Interpersonal difficulties in the treatment of pathological narcissism involve complex dynamics of transference and countertransference constellations (Kernberg 2014). Countertransference can be defined in various ways (Hayes et al. 2018; Kernberg 1965). In the ‘classical’ definition, clinician countertransference is a reaction to the patient's transference, which stir up unconscious conflicts that exist within the clinician and whose reaction typically interferes with the treatment. For example, after needing to cancel a session a patient may accuse the clinician of ‘not really caring’, which may prompt a strong negative emotional reaction in the clinician due to their own need (based on their personal history) to be seen as empathic and compassionate. In contrast, the ‘totalistic’ conception refers to the complete gamut of clinician reactions to the patient, not only those relating to unconscious clinician factors. This conception of countertransference opens up the possibility that there may be something to be learnt about a patient via clinician countertransference. In other words, countertransference may reflect an unconscious communication between a patient and clinician dyad by which the emotional and relational world of the patient is revealed. For instance, in response to a patient who is emotionally and behaviourally volatile and chaotic, a clinician may experience deeper empathy for their patients plight via recognising their own feelings of confusion and disorientation within the session (‘concordant’), or alternatively the clinician may become stern and controlling to try and get the patient to ‘stop being so erratic’ in a manner they learn is strikingly similar to the patients' parents (‘complementary’). In their article, Crisp and Gabbard (2020) include common countertransference reactions towards narcissistic patients, which include feeling chronically excluded, disengaged and detached; feeling ‘pulled’ to empathise and admire; engaging in mutual idealisation; being drawn into supporting and validating the patients vulnerability; feeling envious and competitive; succumbing to patients contempt and devaluation (or alternatively becoming contemptuous themselves); and feeling controlled, scrutinised and monitored. In one of the few empirical explorations of countertransference reactions towards such patients, Tanzilli et al. (2017) report an association between pathological narcissism and clinicians feeling ‘angry/hostile’, ‘criticised/devalued’, ‘helpless/inadequate’ and ‘disengaged’. A further study, distinguishing between subtypes, found grandiosity to be associated with feelings of anger, disengagement and reduced warmth whereas vulnerability was associated with feeling overinvolved/worried (Tanzilli and Gualco 2020). While such emotional reactions towards patients with NPD may partially account for the considerable negative attitudes held towards these patients (Muir et al. 2021; Penney et al. 2017), it is also worth considering the broader cultural attitude held towards individuals perceived as ‘narcissistic’, with inflammatory pejorative language being commonplace across social media (Vorhauer 2024) and even academia (Freestone et al. 2020). However, irrespective of the origin and cultural factors that inform such negative emotional reactions, research specifically exploring attitudes towards patients with narcissistic features is lacking.

The call for dimensional models of personality (Hopwood et al. 2018) has been answered by modern diagnostic systems, whether it be the level of personality functioning described in the *Diagnostic and Statistical Manual of Mental Disorders* (DSM‐5; American Psychiatric Association 2013) and *International Classification of Diseases* (ICD‐11; World Health Organization 2024) or the level of personality organisation in the *Psychodynamic Diagnostic Manual* (PDM‐2; Lingiardi and McWilliams 2017). Within these systems, personality disorders are conceptualised according to (1) the degree of impairment in personality functioning (e.g., No impairment, Personality ‘difficulties’, Mild, Moderate or Severe personality disorder), and (2) relevant personality traits or style (e.g., Negative affectivity, Detachment, Dissociality, Disinhibition, Anankastia), both of which have relevance for clinician countertransference. For instance, it has been identified that different levels of personality disorder severity indicate specific considerations for the quality of the therapeutic alliance and interaction (Bach and Mulder 2022; Bach and Simonsen 2021), where higher functioning narcissistic patients may ‘pacify’ the clinician in the countertransference into unwittingly providing a lengthy but aimless and superficial supportive treatment, however severe presentations may instead seek to unconsciously seek to ‘triumph’ over the clinician in the transference by sabotaging the treatment via self‐destructiveness (Kernberg 2007). Similarly, as these new dimensional systems have a greater capacity to capture the full breadth of narcissistic expression (i.e., grandiosity and vulnerability) as indexed by relevant personality trait domains (e.g., antagonism, negative affectivity; Day et al. 2024) clinician countertransference to seemingly disparate presentations of narcissistic functioning may also hold important diagnostic and clinical information that can assist in the process of personality assessment and intervention.

### 1.1 The Current Study

NPD is not a rare condition in psychiatric settings (Jiang et al. 2019; Kovanicova et al. 2020), with some estimates indicating narcissistic presentations to be as high as 17% in the clinical population (Ronningstam 2009). Nonetheless, narcissism is also known to be a challenging condition to treat, often requiring lengthy psychotherapy engagement and careful navigation of treatment priorities and alliance ruptures (Weinberg and Ronningstam 2020), elsewise risking premature termination (Ellison et al. 2013). Complex transference‐countertransference constellations occurring within the treatment context can disrupt clinician thinking and challenge effective intervention (e.g., Stern et al. 2017) highlighting the need for research that helps elucidate clinician emotional responses and conceptualisation of individuals with narcissistic features. As such, this exploratory study aims to investigate clinician diagnostic ratings and countertransference reactions as identified within open‐ended qualitative responses to either ‘grandiose’ or ‘vulnerable’ hypothetical patients and as extended to similar patients encountered in routine practice. There are no studies that systematically examine clinician countertransference differences between narcissistic subtypes utilising a mixed qualitative and quantitative methods and according to a dimension of severity.

## 2 Method

### 2.1 Participants & Recruitment

Participants were mental health clinicians actively involved in the provision of mental health services to people with a personality disorder. Following institutional ethics board approval, clinicians were recruited using a snowball sampling methodology (Goodman, 1961) via advertisements posted to university institutions, local health facilities and online. Clinicians were offered a 1/16 chance to receive a gift voucher for participating in the research. Clinicians needed to be qualified and be actively involved in the provision of mental health services (e.g., psychologists, psychiatrists, mental health nurses and social workers) in order to participate. All clinicians who completed at least one reflection for one of the vignettes were included in the qualitative analysis in order to capture the maximum possible responses (*N* = 180); however, quantitative comparisons used only clinicians who had completed the whole survey (*N* = 158). Table 1 displays the demographics for the sample.

**TABLE 1:** Clinician demographics (*N* = 180).

Mean age (SD, range)	38.9 (12.0, 22–76)
Mean years clinical experience (SD, range)	11.2 (10.3, 1–50)
Gender	
Female	121 (67.2%)
Male	57 (31.7%)
Non‐binary	2 (1.1%)
Country	
Australia	73 (40.6%)
United Kingdom	59 (32.8%)
Europe	30 (16.7%)
United States	9 (5.0%)
Russia	4 (2.2%)
Canada	3 (1.7%)
Africa	1 (0.6%)
South America	1 (0.6%)
Education	
Postgraduate (masters, PhD or equivalent)	136 (75.6%)
Undergraduate (bachelor, honours or equivalent)	44 (24.4%)
Profession	
Psychologist	66 (36.1%)
Trainee psychologist	60 (31.7%)
Psychiatrist	24 (12.7%)
Other	30 (19.4%)

*Note:* ‘Other’ profession category included the following: clinical nurse consultant (*n* = 1), counsellor (*n* = 3), mental health worker (*n* = 4), occupational therapist (*n* = 3), psychotherapist (*n* = 6), social worker (*n* = 3), or unspecified (*n* = 10).

### 2.2 Measures

#### 2.2.1 Clinical Case Vignettes

Two clinical case vignettes of patients with hypothetical personality disorder presenting with either ‘grandiose’ or ‘vulnerable’ narcissism expressions were presented; however, they were not explicitly labelled as such in the vignette. These vignettes were constructed by Kealy et al. (2017) in consultation with an expert panel who reviewed, amended and endorsed the vignettes as representative of grandiose and vulnerable narcissism regarding central features of narcissistic phenotypic expression (Cain et al. 2008). The vignettes used in this study were slightly modified from their original form in order to remove stereotypically gendered differences in content between male and female versions, but are nonetheless designed to be equivalent in terms of displaying clinical indicators and severity of personality dysfunction, with previous use in exploratory research (Green et al. 2023). All clinicians were presented with both the grandiose and vulnerable vignettes in random order. Clinicians were randomly presented with either the male or female version of the vignettes, with no differences between these versions aside from the pronouns used to describe the hypothetical patient. Vignettes are presented in supplementary materials. After reading each clinical vignette, clinicians responded to the prompt:

Please jot down some brief clinical reflections on this case. This could include your spontaneous reaction, any words or phrases that stand out to you, or your overall general impression.

*Global Personality Disorder Severity*. One question was used to assess global personality disorder severity based on the vignette (“At what degree of overall personality disorder severity would you rate [the patient]?”). Clinicians responded to this question on a scale from 0 (‘no personality disturbance’) to 4 (‘severe personality disturbance’), using the severity classifications in the ICD‐11. Scores of 2 (‘mild personality disorder’) and above indicated a personality disorder. Ratings of personality disorder severity were completed after filling out the qualitative clinical reflection.

#### 2.2.2 Clinician Attitudes

The Attitude to Personality Disorder Questionnaire (APDQ; Bowers and Allan 2006) is a 35‐item scale measuring overall attitudes to personality disorder. In this study, a short 10‐item version was used consisting of items that cover representative positive and negative attitudes towards people with a personality disorder (Day et al. 2018). While negative worded items are typically reversed to provide an overall index of attitudes to personality disorder, in this research, positive and negative items were analysed separately resulting in two separate scales capturing positive (three items) and negative (seven items) sentiment, respectively (and as such items were not reverse scored). Example items include *“I feel frustrated with these patients”* (negative attitude) or *“I feel fondness and affection towards these patients”* (positive attitude). Items are scored on a six‐point Likert scale from ‘1 = Never’ to ‘6 = Always’. Internal consistency of the short APDQ was adequate for positive items (grandiosity: *α* = 0.68, vulnerability: *α* = 0.67) and good for negative items (grandiosity: *α* = 0.87, vulnerability: *α* = 0.84). When completing the measure clinicians were instructed to generalise their ratings beyond just their attitude towards the patient displayed in the vignette (i.e., “please rate how you may feel towards working with patients similar to the vignette based on your clinical experience”).

### 2.3 Procedure

Regarding flow of survey completion, participants were (in this order) (1) presented with a vignette (grandiose/vulnerable), (2) prompted to provide a qualitative clinical reflection, (3) prompted to complete their rating of personality disorder severity and (4) prompted to indicate their attitude towards real patients similar to the vignette they just scored. This process was then repeated in the same order for the corresponding (grandiose/vulnerable) vignette.

### 2.4 Data Analysis Strategy

Qualitative analysis primarily took place by two researchers of the team, one of whom was a doctoral student and trainee psychologist with experience and knowledge in the area of personality disorder, the other was an experienced qualitative researcher and clinical psychologist with specialist expertise in NPD specifically. These two researchers would meet on a regular (i.e., weekly) basis and review analysis progress, with the senior researcher acting as a ‘critical friend’ (Smith and McGannon 2017) discussing and exploring findings and generating possible alternative explanations and interpretations. These refined analyses were then further shared with the wider research team for interpretation and discussion. The current study utilised a critical realist philosophy (Fletcher 2016; Putri et al. 2023), which blends realist ontology (i.e., that an objective reality that exists, separate from human examination) and constructivist epistemology (i.e., that knowledge of phenomena is constructed from and influenced by an individual's experience). That is, we aimed to capture clinician's direct diagnostic impressions and conceptualisations (empirical domain) via identified patterns in clinician responses and across vignettes (actual domain), and we contend that there are underlying mechanisms that inform these identified patterns (real domain)—one such underlying mechanism being countertransference.

Researchers first began by reading and re‐reading all participant responses to gain familiarity with the data, highlighting and notating salient comments and meeting to discuss initial impressions and reactions. Clinician qualitative responses were then analysed via two different methodologies of thematic analysis (Braun and Clarke 2006, 2020) to provide a comprehensive analysis. First, ‘codebook’ thematic analysis was conducted (Braun et al. 2019) in order to capture clinician diagnostic conceptualisations. In this methodology, DSM‐5 diagnostic categories were pre‐selected in advance to create a thematic ‘codebook’. Clinician responses were then read with a specific focus on identifying any instance where clinicians referenced these pre‐generated codes. This method is a way of quantifying clinician qualitative responses and generating frequencies, as any time clinicians spontaneously referenced a psychiatric term it was allocated to the corresponding diagnostic category (e.g., clinicians who use the terms “depressed/depression/major depression/depressive symptoms” and similar was coded under the category ‘Depressive disorders’). The second approach was to use ‘reflexive’ thematic analysis (Braun et al. 2019), a fully qualitative approach where themes are conceptualised as meaning‐based patterns. Coding takes place in an organic and iterative process in which the researchers aim to provide a coherent interpretation of the data (rather than simply to summarise). As such, identified concepts and codes were free to be split, renamed or combined with other codes over the course of the analysis as reflecting the researchers ongoing conceptualisation of the data. Final codes and concepts were the result of the primary researchers meeting, discussing results and, in conjunction with the wider research team, agreeing on the output as reflecting the best conceptualisation of the data.

Quantitative analysis included conducting means comparisons (repeated measures *t*‐tests) between severity ratings and clinician attitude scores (positive/negative) towards narcissistic grandiosity and vulnerability. Correlation analyses were also conducted to explore associations between clinician attitude scores and ratings of personality disorder severity. Cohen's *d* was used as an estimate of effect size, utilising standard interpretation benchmarks for magnitude of difference (Cohen 1988; Lovakov and Agadullina 2021).

## 3 Results

### 3.1 Diagnostic Ratings and Clinical Conceptualisation

The majority of participants made diagnoses in their clinical reflection for both grandiose (*n* = 133) and vulnerable (*n* = 132) vignettes, with clinicians typically only using one or two psychiatric terms per response. However, there were differences in the diagnostic terms used by participants between the two vignettes.

**FIGURE 1:**
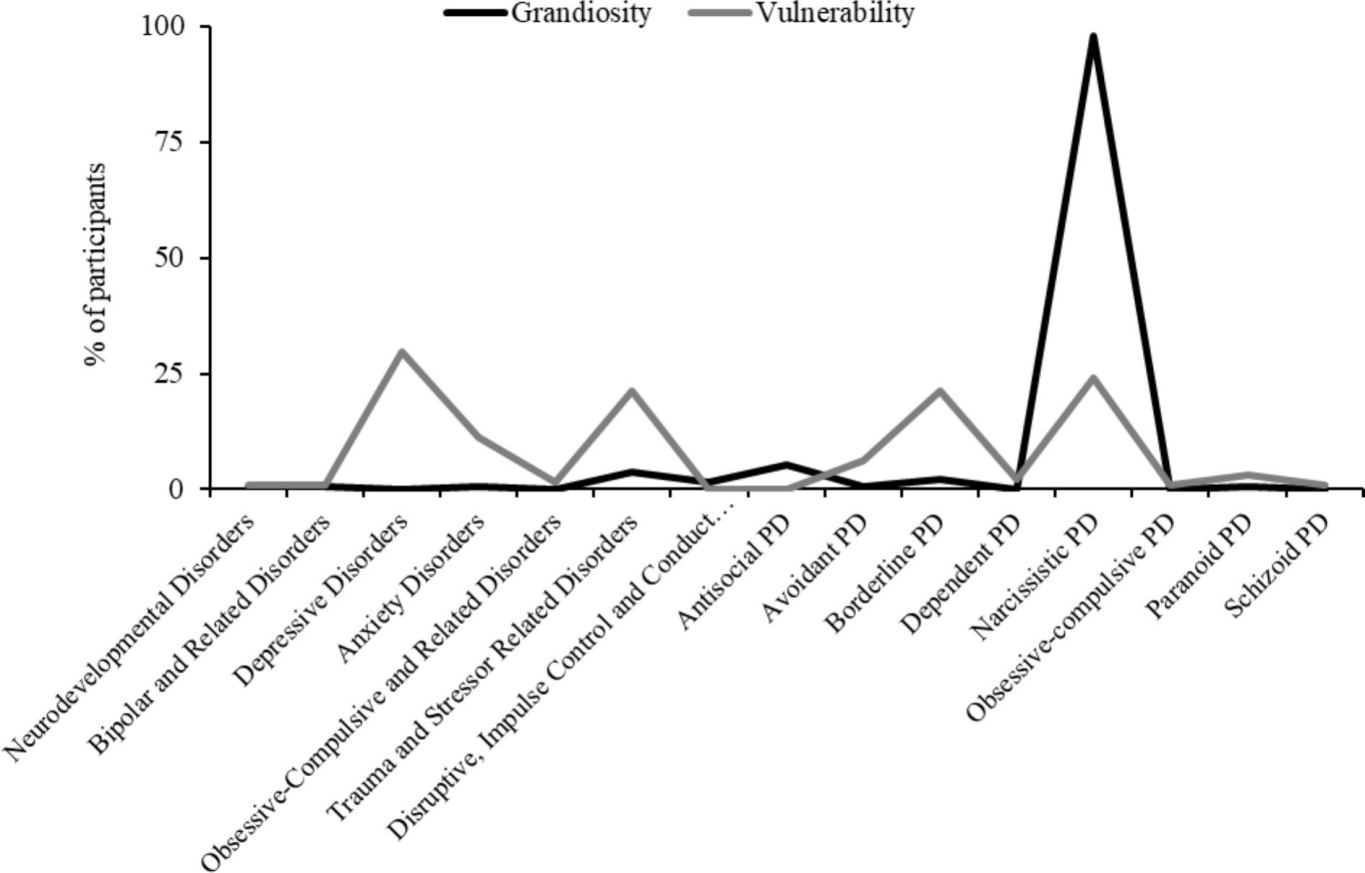
Clinician diagnoses for the narcissistic grandiosity (*n* = 133) and vulnerability (*n* = 132) vignettes.

The grandiose vignette was almost exclusively conceptualised using phrases related to ‘NPD’ (97.7% of participants), e.g., “[the patient] appears to be presenting with a narcissistic personality structure characterized by persistent difficulties in a range of interpersonal domains, underpinned by experiences of entitlement, superiority, dismissiveness of others, grandiose fantasies and a grandiose sense of self” (#107). Whereas the vulnerable vignette was more variably conceptualised across multiple different diagnostic terms, particularly ‘depressive disorders’ (29.6%), ‘anxiety disorders’ (11.4%), ‘trauma and stressor‐related disorders’ (21.2%), ‘borderline personality disorder’ (21.2%) and ‘NPD’ (24.2%). E.g., “Some symptoms indicate BPD such as chronic feelings of emptiness, idealisation/devaluation, stress‐induced dissociation and paranoia, abandonment fears. However, these symptoms could also be indicated of CPTSD ‐ a negative self‐concept and anticipates people will exploit/betray her … [also] vulnerable narcissism ‐ there is some sense of resenting others for not being admired and sense of paranoia that others want to exploit her” (#91). The frequency of coded diagnostic terms for both grandiosity and vulnerability are presented in Figure 1.

### 3.2 Emotional Reactions and Countertransference

Clinicians regularly shared their emotional reaction towards the patient depicted in the vignettes, as well as their general emotional reactions towards patients in their actual clinical practice that were similar to that of the vignette.

For the narcissistic grandiosity vignette, this typically included feelings of hostility, disconnection and inadequacy, whereas the narcissistic vulnerability vignette most commonly evoked feelings of sadness and sympathy. Interestingly, clinicians also described feeling vague negative feelings or uneasiness towards the narcissistic vulnerability case. Key concepts relating to clinician emotional reactions, example statements and formulated meanings for narcissistic grandiosity and vulnerability are presented in Tables 2 and 3, respectively.

**TABLE 2:** Clinician emotional reactions towards the narcissistic grandiosity vignette.

Concept	Example statements	Formulated meaning
**Anger–annoyance**	*“I think I could also become frustrated with him. If I felt pressured by him or if I got the impression he is ‘entitled’ to something”* (#177) *“My immediate reaction is anger at his infidelity and that he has a sense of injustice when he is the one acting immorally”* (#136) *“My first reaction is a sense of distaste towards [the patient] … my spontaneous reaction is that I would not want to work with her”* (#12)	Patient's grandiosity, challenging behaviours and lack of insight off‐putting to clinicians, inspiring hostile countertransference reactions.
**Non‐identification, lack of empathy**	*“I feel strongly for [the patients] wife, and worry about her needs being met in this relationship … I find it hard with this type of presentation to return focus to the client”* (#40) *“I feel sorry for people surrounding him.”* (#151)	Clinicians are alienated from ‘joining’ the internal world of the patient. Instead, clinicians are pulled to identify and empathise with others.
**Hopeless–inadequate**	*“[I feel] some hopelessness … he sounds tricky and I'm not sure what will help”* (#136) *“Does not feel like therapy will be useful”* (#154) *“I would probably refer out to local experts in personality”* (#96) *“I think he would not last with me, I do not reflect enough prestige”* (#27)	Clinicians feel inept, that their skills are unable to adequately address the needs of the patient.

**TABLE 3:** Clinician emotional reactions towards the narcissistic vulnerability vignette.

Concept	Example statements	Formulated meaning
**Sadness, sympathy–empathy**	*“I felt for this man. He seems to be struggling with the vicissitudes of life”* (#135) *“This is a very sad state of being for [this patient]”* (#27) *“I initially felt bad for [this patient]. That she's got such a vulnerable core that her lack of boundaries/identity is exploding out of her”* (#4)	Patient's overt suffering and vulnerability elicits empathy and concern from clinicians.
**Feeling uneasy, doer‐and‐done‐to**	*“A spontaneous reaction was some frustration at [this patient]’s apparent martyr‐like behaviours”* (#9) *“I experienced a sense of nervousness and hopelessness [about the patients] ‘long standing’ difficulties … I also experienced this when I heard of their “secret pride”* (#39) *“If I was to see this person I imagine I would feel quite uncomfortable in being placed in the ‘expert’ position”* (#71)	Clinicians identify or anticipate feeling a vague negative feeling related to a deeper issue within the patient, not yet explicitly manifest. A feeling that ‘something is not quite right’.

### 3.3 Personality Disorder Severity and Clinician Attitudes

Regarding clinician ratings of personality disorder severity, the grandiose vignette was rated as displaying more severe personality impairment than the vulnerable vignette, despite them being constructed to provide identical severity markers. As such, while most clinicians endorsed a severity rating for both vignettes consistent with a diagnosis of personality disorder (‘2’ or higher), this occurred 88% of the time for the grandiose vignette, but 75% for the vulnerable vignette. Regarding clinician attitudes, clinicians felt significantly more negative towards narcissistic grandiosity compared to narcissistic vulnerability, with the highest scored negative items towards both grandiosity and vulnerability being feeling ‘frustrated’ and ‘drained’. Similarly, clinicians felt significantly more positive towards narcissistic vulnerability than towards narcissistic grandiosity, with the highest scored positive items towards vulnerability being feeling ‘protective’ and ‘fondness’; however, notably, clinicians rated feeling ‘interested’ approximately equally for both grandiosity and vulnerability. Observed differences in clinician ratings for personality disorder severity and attitudes were statistically significant, as presented in Table 4. Effect sizes were ‘medium’ for ratings of personality disorder severity, indicating meaningful discrepancies between clinicians' judgement of impairment between narcissistic grandiosity and vulnerability. However, effect sizes for clinician attitude scores were ‘large’ between narcissism subtypes, indicating substantial variation in emotional responses (both positive and negative) between grandiosity and vulnerability.

**TABLE 4:** Comparing clinician ratings between narcissistic grandiosity and vulnerability.

Clinician rating	M (SD)	*t*	*p*	*d*
Personality disorder severity		8.1	< 0.001	0.6
Grandiose	2.7 (0.9)			
Vulnerable	2.2 (0.9)			
Positive attitudes		9.8	< 0.001	0.8
Grandiose	3 (0.8)			
Vulnerable	3.6 (0.7)			
Negative attitudes		11	< 0.001	0.9
Grandiose	3 (0.8)			
Vulnerable	2.4 (0.7)			

*Note:* Cohen's *d* effect size interpretation: small = 0.2, medium = 0.5, large = 0.8 (Lovakov and Agadullina 2021).

**FIGURE 2:**
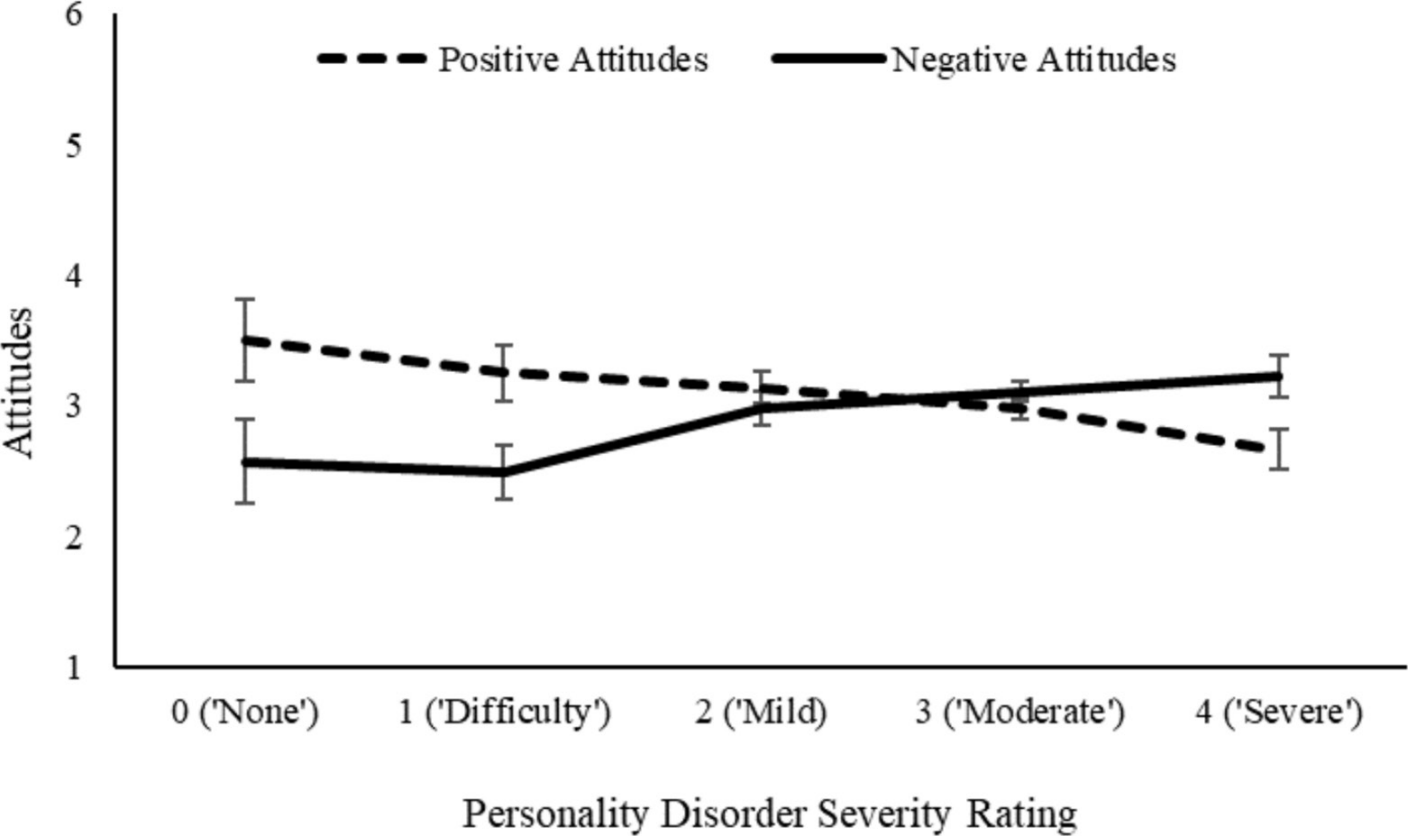
Attitudes towards narcissistic grandiosity indexed as a function of personality disorder severity ratings.

**FIGURE 3:**
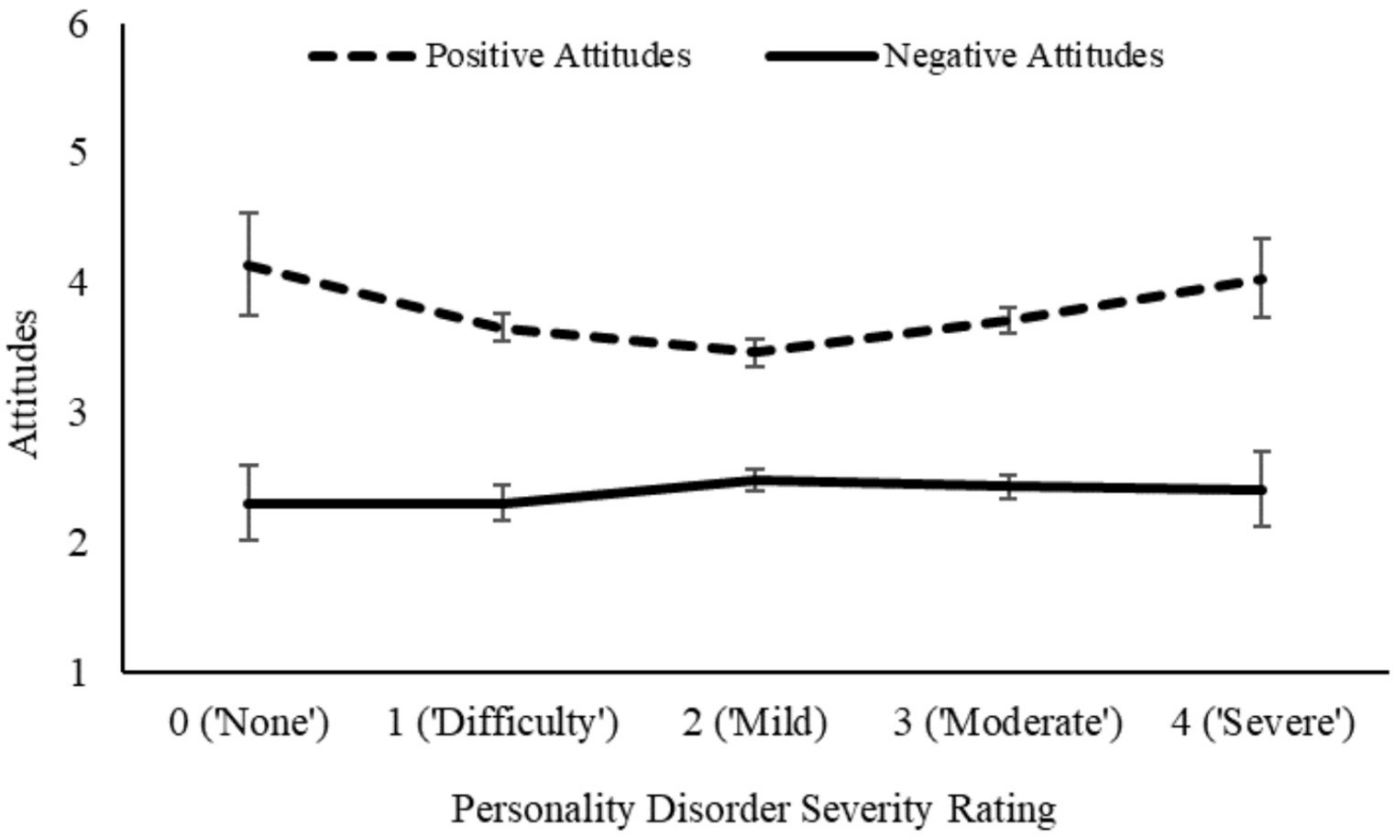
Attitudes towards narcissistic vulnerability indexed as a function of personality disorder severity ratings.

Correlation analysis, displayed in Table 5, indicated a strong positive correlation between ratings of personality disorder severity for both grandiosity and vulnerability, suggesting that clinicians were broadly consistent in their ratings between vignettes. However, only narcissistic grandiosity showed a relationship with attitudes, as more negative (and less positive) attitudes showed a significant association with ratings of personality disorder severity, whereas there was no such association for vulnerability. The relationship between severity ratings and attitudes are displayed in Figures 2 and 3.

**TABLE 5:** Correlation matrix of personality disorder severity ratings and clinician attitudes.

Variable	M (SD)	1.	2.	3.	4.
1. Personality disorder severity (grandiosity)	2.7 (0.9)	1	0.60**	−0.23**	0.25**
2. Personality disorder severity (vulnerability)	2.2 (1.0)	—	1	0.02	0.05
3. Positive attitudes	3.3 (0.7)	—	—	1	−0.24**
4. Negative attitudes	2.7 (0.7)	—	—	—	1

* *p* = < 0.05.

** *p* = < 0.01.

## 4 Discussion

This study sought to explore clinicians' conceptualisation and countertransference towards differing expressions of narcissistic functioning. Clinicians read vignettes of both ‘grandiose’ and ‘vulnerable’ narcissism expressions and completed a rating of personality disorder severity of the patient depicted in the vignette. Clinicians then provided a qualitative reflection and completed a rating of attitudes towards patients in their clinical practice that are similar to that presented in the vignette. Narcissistic grandiosity was readily identified by clinicians as resembling prototypical features of NPD as per DSM‐5 categorical criteria and rated the grandiose vignette as displaying moderate to severe impairment in personality functioning. Clinicians also described negative countertransference such as feeling anger/annoyance, lack of empathy and inadequacy in their qualitative responses. Conversely, narcissistic vulnerability was variably conceptualised across multiple diagnostic categories including depressive disorders, borderline personality disorder and NPD, the vulnerable vignette was also rated as less severe in terms of personality impairment. Countertransference statements included feeling sadness and empathy, but also a vague feeling of unease or discomfort towards the patient. Comparing attitude scores, clinicians felt significantly more positive and less negative towards the vulnerable vignette than compared to the grandiose vignette. Correlation analysis indicated that clinician negative attitudes towards narcissistic grandiosity were associated with more severe ratings of personality impairment.

There are two potential interpretations of these findings. The first is that when rating the severity of personality dysfunction for the grandiose vignette, clinicians' negative feelings may have interfered with accurate appraisal of functioning such that those who had a stronger negative attitude overestimated the pathology of the patient due to their negative bias towards them. This ‘stigma’ interpretation is supported by clinician qualitative responses, which described hostile feelings, subsequent empathy deficits and state a lack of desire to work with such patients. Indeed, NPD has been described as an inordinately stigmatised condition (Koepernik et al. 2022; Penney et al. 2017), where the term itself (‘narcissist’) doubles as both a diagnosis and a pejorative insult. Such negative attitudes may stem from the intense negative countertransference and interpersonal patterns that occur as reported by both clinicians (Crisp and Gabbard 2020; Tanzilli and Gualco 2020; Tanzilli et al. 2017) and intimate partners and family (Day et al. 2019; Day et al. 2021, 2022). This kind of negativistic attitude towards narcissistic grandiosity is why treatment guidelines stress finding ways of not acting out negative countertransference and instead to find ways of establishing a collaborative alliance in the treatment (Weinberg and Ronningstam 2020). Similarly, research has highlighted the need to emphasise the ‘vulnerable core’ of narcissistic disorders in order to increase empathy and likability of these patients (Andino et al. 2022; Koepernik et al. 2022; Stanton et al. 2018). Our results also support this notion, as clinicians rated the vulnerable dimension of narcissism with more positive attitudes, as well as qualitatively described more sympathy and empathic connection towards them.

However, an alternative interpretation can also be considered using this same data that suggests the precise opposite. That is, that clinicians' negative countertransference towards narcissistic grandiosity served as a useful cue regarding the pathology of the underlying personality disorder, as shown by providing ‘correct’ diagnoses and the association between severity and attitude ratings. Whereas it seems clinicians were not able to use their countertransference in the same way with narcissistic vulnerability (and may have even been misled by it). Despite the vignette displaying the necessary clinical indicators, the non‐identification rate of a personality disorder for the vulnerable vignette was over double that of the grandiose vignette. Clearly, through both attitude ratings and qualitative responses, participants felt sympathy and empathy for the vulnerable patient—however, Crisp and Gabbard (2020) caution against this kind of countertransference, termed ‘identification with vulnerability’ (p. 151), whereby clinicians are drawn into a positive countertransference due to the patients desire to be loved and admired. Indeed, empirical research reports an ‘overinvolved/worried’ subtype in response to narcissistic vulnerability (Tanzilli and Gualco 2020) that is consistent with the findings reported here. As such, this ‘collusion’ interpretation raises the suggestion that a positive countertransference is not necessarily a therapeutic one, and can result in a stagnant treatment that remains superficial for long periods without advancing (Caligor et al. 2018).

While these two interpretations of the data, alternating along lines of ‘stigma’ or ‘collusion’, may seemingly be at odds with each other—our view is that both capture the different challenges of working effectively with patients with narcissistic pathology. Overall, these results highlight the importance of attending to countertransference (either positive or negative) in assessing and formulating a patient's personality style. While certain therapeutic approaches consider transference constellations a central component of assessment and conceptualisation (e.g., transference focused psychotherapy), modalities of different theoretical backgrounds have also found specifically attending to transference a useful clinical aid (e.g., cognitive behavioural therapy; Moorey 2014; Prasko et al. 2010). Indeed, research indicates effective countertransference management as a key factor in successful psychotherapy outcome (Hayes et al. 2018), which unexamined may result in boundary violations, combativeness and ultimately termination of therapy (Hayes et al. 2015). Overall, the findings of this study are consistent with recommendations for treating narcissistic pathology, balancing these two countertransference dynamics—to neither “respond defensively, aggressively, or dismissively to the narcissistic patient” or to “withdraw and collude with the patient's denial of pathology” (Caligor et al. 2015, p. 420).

### 4.1 Limitations

The current study has a number of limitations, which should be taken into consideration when interpreting the results presented. First, while the reliance on vignette‐based methodology allows for a high degree of control, it may not fully capture the complexities of real‐world psychotherapy regarding both narcissistic presentation (i.e., individuals presenting in much more mixed or fluctuating grandiosity‐vulnerability constellations) and counter‐transferential reactions. Future research should extend these findings by analysing real psychotherapy sessions, using observational methods (e.g., via a third‐party), clinician–client transcripts, or methods like ecological momentary assessment to assess how the studied variables manifest in real time. Second, the use of a brief attitude measure (i.e., APDQ) for indexing countertransference reactions places some limits the interpretability of findings, as this measure is necessarily broad and non‐specific. While this was suitable for the current study, given the methodological design and paired with clinician qualitative responses, future research seeking to expand on these results could utilise a more robust approach. For instance, utilising well established measures that specifically assess countertransference within therapeutic contexts (e.g., Therapist Response Questionnaire; Tanzilli et al. 2016) or engaging in in‐depth interviews capturing moment‐to‐moment narrative recollections (e.g., Core Conflictual Relationship Themes; Luborsky and Crits‐Christoph 1998) of clinicians working with patients with narcissistic pathology will likely provide results of more fine grain detail and is a suggestion for future research.

### 4.2 Clinical Implications for Diagnosis and Treatment

Although this study focused on the two phenotypic themes of narcissistic grandiosity and narcissistic vulnerability to highlight differences in clinicians' countertransference, both expressions should be considered in the clinical context as different manifestations of the same disorder rather than discrete forms of pathological narcissism. In other words, when working with patients with pathological narcissism, instances of both grandiosity *and* vulnerability are bound to be present within the same individual, often shifting between both expressions as a result of their vacillating sense of self and incessant effort to protect their self‐esteem (Kernberg 1985; Pincus et al. 2014; Ronningstam 2009). Therefore, the contrasting countertransference responses towards grandiose and vulnerable expressions of narcissism identified in this study, further highlight the following: (1) the complexity of working with these patients (i.e., dealing with the resulting confusion of experiencing both sadness and empathy while also feeling anger and annoyance), as well as (2) the potential risk of minimising the grandiosity underlying the narcissistic patients who initially present as more vulnerable. Indeed, although the covertly narcissistic patient may present as shy, depressed and inhibited, closer contact will likely reveal exhibitionistic and grandiose fantasies (Levy 2012). Moreover, despite seemingly contrasting expressions, clinicians should keep in mind that both vulnerable and grandiose presentations share attitudes of being special and entitled to special treatment (Jauk et al. 2017; Krizan and Herlache 2017). The risk of minimising the vulnerable patient's grandiosity is particularly important considering that treatment‐seeking narcissistic patients more often initiate contact with clinicians when they are in a more symptomatic, vulnerable state, with the grandiosity typically emerging later as the therapeutic relationship develops (Ellison et al. 2013). Although countertransference reactions have been found to be helpful in diagnosing pathological narcissism (Gabbard 2009), countertransference can also lead clinicians to misperceive their patients and thus inaccurately diagnose or conceptualise their cases (Hayes et al. 2015). Considering that such risk may be increased in the presence of vulnerable patients – as seen in the current study with the tendency to minimise the severity of their personality dysfunction and to initially experience greater empathy and sadness in their presence – clinicians may reduce the risk of misdiagnosis by being attentive, in particular, to the feeling of ‘walking on thin ice’ and feeling fearful of wounding a delicate and hypersensitive patient, combined with other contrasting reactions that unfold in prolonged contact with such patients (including the feelings of incompetence, resentment and annoyance that usually accompany the shift towards a grandiose state).

Regarding technical and treatment implications, strong countertransference reactions may indicate appropriate therapeutic interventions. For instance, upon recognising the feeling of humiliation and inadequacy following what is felt to be a severe devaluation from a patient, a clinician may recognise the way in which this ‘concordant’ countertransference may provide a window into the depth of the patients own self‐loathing in what otherwise may seem to be an entitled and grandiose character. Armed with this knowledge, the clinician avoids responding in a ‘like for like’ manner, despite what may feel an overwhelming impulse to “point out suddenly and devastatingly that the patient has earned little … and deserves little” as this would be a complete “assault on the very psychological foundations” that may hold a very fragile patient together (Groves 1978, p.885). Instead, the therapist may respond with curiosity, authentically acknowledge their own realistic shortcomings, and provide an opportunity to validate feelings of disappointment of the patient (which may be linked to intense, unconscious shameful dependency strivings). Simultaneously, the therapist may also demonstrate that while not being ‘perfect’, they maintain a steadfast belief that they have a legitimate expertise and capacity to offer something meaningful to the patient in psychotherapy. Such moments in the treatment reflect what some have described as transference ‘tests’ (Gazzillo et al. 2022) where the therapist is placed to “act in either a re‐traumatising or transformative way” (p. 210). In doing so, the therapist also models an important skill which the patient may be seeking to implicitly learn about. That is, transference tests may be a way for the patient to unconsciously explore more flexible solutions to inter‐intrapersonal dilemmas they themselves have been unable to solve. I.e., how does the clinician handle being shamed, without retreating into their own narcissistic dynamics of either counter‐attack or total collapse? Helping a patient with narcissistic dynamics navigate such oscillations between grandiose and vulnerable states in the pursuit of a ‘good enough’ sense of self and self‐esteem is a key treatment task and, if successful, a major achievement in the therapy.

In sum, recognising the clinical phenomenology of both narcissistic vulnerability and grandiosity, and their impact on the clinicians mixed countertransference reactions, can improve the clinical utility of diagnosing pathological narcissism and reduce risks of misdiagnosis and subsequently maladapted treatment choices.

## Ethics Statement

The study received ethical approval from the Institutional Review Board (ETH2223‐1126) from City, University of London.

## Consent

Participants provided informed consent prior to participating.

## Conflicts of Interest

The authors declare no conflicts of interest.

## Supporting information


**Data S1.** Supporting Information

## Data Availability

The datasets generated and/or analysed during this study can be obtained from the corresponding author on reasonable request.
