# Supplementary material for: Clinician Diagnostic Ratings and Countertransference Reactions Towards Grandiose and Vulnerable Narcissism
Source: Clin Psychol Psychother. 2025 Mar 27;32(2):e70070. doi: 10.1002/cpp.70070 (PMC11949600; doi:10.1002/cpp.70070)
Supplement: Supplementary file 1 — Data S1. Supporting Information [file CPP-32-e70070-s001.docx]

Supplementary Material

**Grandiose Vignette – Male (Adapted from** **Kealy et al., 2017)**

**Patient 1: Mr. G.**

Mr. G. is a 35-year-old man who is seeking psychotherapy at the request of his wife due to longstanding relationship difficulties. His wife issued an ultimatum that he obtain treatment or else she would end the marriage. Mr. G. reports that their frequent conflicts are due, in his view, to his wife’s lack of respect for him and refusal to comply with what he wants her to do. He indicates that he has a superior intelligence to his wife, and for that matter, to most people he meets. He reasons that others should simply abide by his instructions.

Mr. G. acknowledges becoming angry when people don’t show enough respect for him; he feels that he regularly outperforms his co-workers, yet his efforts are not admired. In fact, he feels that other people – whether at work or at the gym – tend to envy his abilities and personal attributes. He reasons that this is why he has been held back from a position of more importance at his firm. He often imagines himself taking over the company and firing everyone who has been “disrespectful” to him over the years. He has an alternate fantasy of leaving his firm and becoming a professional athlete – he feels certain he could do this if only his wife would cease restricting his trips to the gym.

Mr. G. acknowledges that he has had extramarital affairs, which his wife is unaware of. He feels that he should be entitled to these liaisons, although he knows his wife would feel hurt and would end the marriage if she found out about them.

He expresses little enthusiasm for engaging in psychotherapy – he doubts whether any therapist would have much to offer him – but he wants to maintain his marriage and find a way to be less irritated by others.

**Vulnerable Vignette – Male (Adapted from Kealy et al., 2017)**

**Patient 2: Mr. V.**

Mr. V. is a 42-year-old man who is seeking psychotherapy to address longstanding feelings of depression and anxiety. He indicates that for most of his life he has struggled with chronic feelings of emptiness and a sense of being profoundly lost and alone. He feels this way in spite of being married and having two children. He sometimes wonders what his family sees in him, and that unless he does enough to support the family, they would likely turn against him.

In addition to longstanding depressive feelings, Mr. V. reports experiencing pronounced dysphoria if someone slights him or rejects him in any way. In fact, he often anticipates being rejected, and consequently feels a need to constantly prove himself to others. Among colleagues at his work he feels himself to be “a little boy among men” in that he regards others as being more articulate and capable than himself. He often ruminates on events that have occurred which confirm his inferiority, and he tends to dwell on experiences which, in his view, produced a profound sense of humiliation.

Mr. V. reports having few close friends and few activities that he allows himself to engage in for pleasure; he devotes his time instead to avoiding the disapproval of others. He feels he works harder than anyone else he knows, but he reasons that he must do so simply to keep up with others and avoid being shamefully exposed as a fraud. At the same time, he acknowledges that he takes secret pride in being more industrious than others, and he resents not being admired for his diligence. He doesn’t believe that his wife understands him. Rather, he feels that she exploits his sensitivities and tries to make him feel inferior and weak, which results in painful depressive affect along with urges to flee the marriage. He fantasizes that an ideal partner would provide a perfect, transcendent love that would wash away his negative feelings.

Mr. V. approaches psychotherapy cautiously, regarding the therapist as an expert authority figure who might also seek to make him feel bad.

**Grandiose Vignette – Female (Adapted from Kealy et al., 2017)**

**Patient 1: Mrs. G.**

Mrs. G. is a 35-year-old woman who is seeking psychotherapy at the request of her husband due to longstanding relationship difficulties. Her husband issued an ultimatum that she obtain treatment or else he would end the marriage. Mrs. G. reports that their frequent conflicts are due, in her view, to her husband’s lack of respect for her and refusal to comply with what she wants him to do. She indicates that she has a superior intelligence to her husband, and for that matter, to most people she meets. She reasons that others should simply abide by her instructions.

Mrs. G. acknowledges becoming angry when people don’t show enough respect for her; she feels that she regularly outperforms her co-workers, yet her efforts are not admired. In fact, she feels that other people – whether at work or at the gym– tend to envy her abilities and personal attributes. She reasons that this is why she has been held back from a position of more importance at her firm. She often imagines herself taking over the company and firing everyone who has been “disrespectful” to her over the years. She has an alternate fantasy of leaving her firm and becoming a professional athlete – she feels certain she could do this if only her husband would cease restricting her trips to the gym.

Mrs. G. acknowledges that she has had extramarital affairs, which her husband is unaware of. She feels that she should be entitled to these liaisons, although she knows her husband would feel hurt and would end the marriage if he found out about them.

She expresses little enthusiasm for engaging in psychotherapy – she doubts whether any therapist would have much to offer her – but she wants to maintain her marriage and find a way to be less irritated by others.

**Vulnerable Vignette – Female (Adapted from Kealy et al., 2017)**

**Patient 2: Mrs. V.**

Mrs. V. is a 42-year-old woman who is seeking psychotherapy to address longstanding feelings of depression and anxiety. She indicates that for most of her life she has struggled with chronic feelings of emptiness and a sense of being profoundly lost and alone. She feels this way despite being married and having two children. She sometimes wonders what her family sees in her, and that unless she does enough to support the family, they would likely turn against her.

In addition to longstanding depressive feelings, Mrs. V. reports experiencing pronounced dysphoria if someone slights her or rejects her in any way. In fact, she often anticipates being rejected, and consequently feels a need to constantly prove herself to others. Among colleagues at her work she feels herself to be “a little girl among women” in that she regards others as being more articulate and capable than herself. She often ruminates on events that have occurred which confirm her inferiority, and she tends to dwell on experiences which, in her view, produced a profound sense of humiliation.

Mrs. V. reports having few close friends and few activities that she allows herself to engage in for pleasure; she devotes her time instead to avoiding the disapproval of others. She feels he works harder than anyone else she knows, but she reasons that she must do so simply to keep up with others and avoid being shamefully exposed as a fraud. At the same time, she acknowledges that she takes secret pride in being more industrious than others, and she resents not being admired for her diligence. She doesn’t believe that her husband understands her. Rather, she feels that he exploits her sensitivities and tries to make her feel inferior and weak, which results in painful depressive affect along with urges to flee the marriage. She fantasizes that an ideal partner would provide a perfect, transcendent love that would wash away her negative feelings.

Mrs. V. approaches psychotherapy cautiously, regarding the therapist as an expert authority figure who might also seek to make her feel bad.

Reference

Kealy, D., Goodman, G., Rasmussen, B., Weideman, R., & Ogrodniczuk, J. S. (2017). Therapists’ perspectives on optimal treatment for pathological narcissism. *Personality Disorders: Theory, Research, and Treatment, 8*, 35–45. https://doi.org/10.1037/per0000164
